# Crystal structure of the co-crystal *fac*-tri­aqua­tris(thio­cyanato-κ*N*)iron(III)–2,3-di­methyl­pyrazine (1/3)

**DOI:** 10.1107/S2056989015004831

**Published:** 2015-03-18

**Authors:** Olesia I. Kucheriv, Sergii I. Shylin, Tetiana A. Ilina, Sebastian Dechert, Il’ya A. Gural’skiy

**Affiliations:** a Department of Chemistry, Taras Shevchenko National University of Kyiv, Volodymyrska st. 64, Kyiv 01601, Ukraine; b Kherson National Technical University, Beryslavske st. 24, Kherson 73008, Ukraine; c Institute of Inorganic Chemistry, Georg-August-University Göttingen, Tammannstrasse 4, Göttingen D-37077, Germany

**Keywords:** crystal structure, Fe^III^ complex, iso­thio­cyanate ligand, pyrazine, co-crystal, hydrogen bonding

## Abstract

In the crystal of the title compound, [Fe(NCS)_3_(H_2_O)_3_]·3C_6_H_8_N_2_, the Fe^III^ cation is located on a threefold rotation axis and is coordinated by three N atoms of the thiocyanate anions and three water mol­ecules in a *fac* arrangement, forming a slightly distorted N_3_O_3_ octa­hedron. Stabilization within the crystal structure is provided by O—H⋯N hydrogen bonds; the H atoms from coordinating water mol­ecules act as donors to the N atoms of guest 2,3-di­methyl­pyrazine mol­ecules, leading to a three-dimensional supra­molecular framework.

## Abstract 2

The Fe^III^ complex located on a threefold rotation axis links with di­methyl­perazine mol­ecules *via* O—H⋯N hydrogen bonds,forming a three-dimensional supra­molecular framework.

## Chemical context   

In the large family of coordination compounds, materials showing a tunable character of their physical properties (*e.g.*, electrical, magnetic, optical *etc*) are of special inter­est. Attempts to design compounds with such tunability have revealed the possibility to target the property of inter­est through the rational choice of ligands in transition metal complexes. For instance, variation of the aromatic *N*-donor ligand can lead to possible spin-state modulation of transition metals. In certain cases, these complexes can even possess spin crossover behaviour (transition between low and high spin states of a metal). The phenomenon of spin transition, which is one of the most known examples of mol­ecular bis­tability, can be provoked by some external stimuli (temperature, pressure, light, magnetic field, absorption of some compounds) and is followed by a change of the optical, magnetic and electric properties (Gütlich & Goodwin, 2004 ▸). 
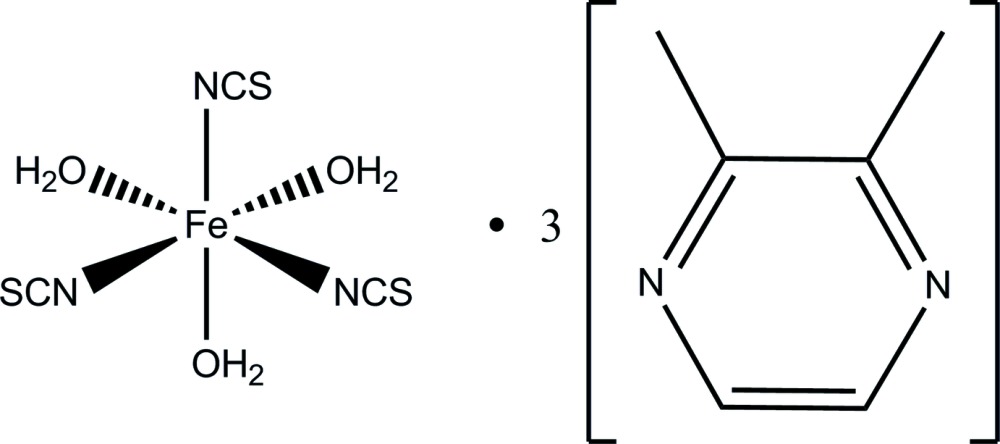


One of the simplest bridging *N*-donor ligands in the design of coordination polymers is pyrazine. This ligand is known for the formation of not only low-dimensional chains and sheets but also of some more complicated architectures, such as [Ag(pz)](CB_11_H_12_) [CB_11_H_12_^−^ is the monocarba-*closo*-dodeca­borate(−) anion], which exhibits a three-dimensional structure made up of checkerboard sheets of silver cations and anions connected by pillars of bridging pyrazine ligands (Cunha-Silva *et al.*, 2006 ▸). In addition, pyrazine is able to construct Hofmann clathrates – spin crossover compounds with general formula [Fe^II^ *M* ^II^ (pz)(CN)_4_]_∞_ where *M* = Ni, Pd or Pt (Niel *et al.*, 2001 ▸). A combination of pyrazine ligands with thiocyanates instead of tetracyanidometalates leads to the two-dimensional coordin­ation polymer [Fe(pz)_2_(NCS)_2_]_∞_ with an anti­ferromagnetic exchange between the metal cations (Real *et al.*, 1991 ▸). In this context, we attempted to synthesize an Fe^II^ thio­cyanate complex with 2,3-di­methyl­pyrazine; however, the exposure of the starting material [Fe(OTs)_2_]·6H_2_O (OTs = *p*-toluene­sulfonate) to the oxygen in the air led to the oxidation of Fe^II^ and to the formation of the title compound.

## Structural commentary   

**Figure 1:**
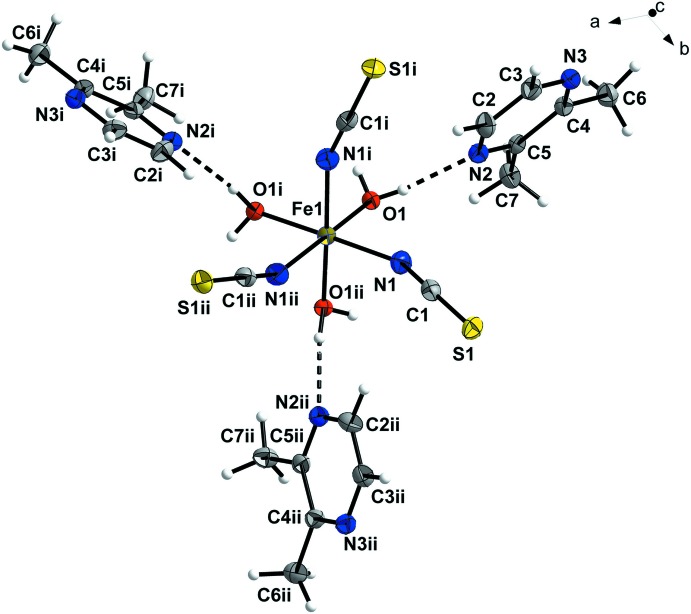
The mol­ecular structure of the title compound, with displacement ellipsoids drawn at the 50% probability level. [Symmetry codes: (i) −*y* + 1, *x* − *y* + 1, *z*; (ii) −*x* + *y*, −*x* + 1, *z*.]

In the crystal structure of the title compound, the Fe^III^ cation is located on a threefold rotation axis and is in an octa­hedral coordination environment formed by three N atoms of the thio­cyanate anions and three O atoms of water mol­ecules arranged in a *fac* configuration (Fig. 1 ▸). The distance between the Fe^III^ ion and the N atoms [2.025 (4) Å] is longer than that between the Fe^III^ ion and the O atoms [2.034 (3) Å] and therefore the FeN_3_O_3_ octa­hedron is slightly distorted. These structural features are typical for related compounds (Shylin *et al.*, 2013 ▸, 2015 ▸). The thio­cyanate ligands are bound through nitro­gen atoms and are quasi-linear [N1—C1—S1 = 179.5 (4)°], while the Fe–NCS linkages are bent [C1—N1—Fe1 = 157.0 (4)°]. Previously reported complexes with an N-bound NCS group possess similar structural features (Petrusenko *et al.*, 1997 ▸).

## Supra­molecular features   

**Figure 2:**
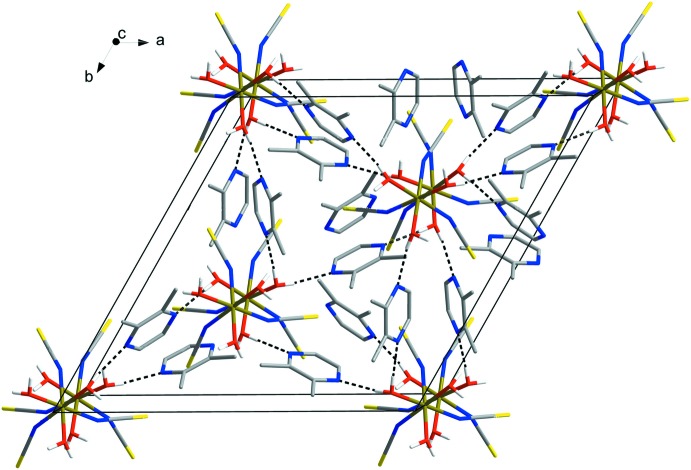
Crystal structure of the title compound, showing hydrogen bonds as dashed lines. H atoms not involved in hydrogen bonding have been omitted for clarity. Colour key: bronze Fe, yellow S, blue N, grey C and red O.

In the title compound, the crystal packing is stabilized by O—H⋯N hydrogen bonds (Table 1 ▸): the H atoms from coordin­ating water mol­ecules act as donors to the N atoms of guest 2,3-di­methyl­pyrazine mol­ecules. The compound contains three guest mol­ecules of pyrazine per Fe^III^ cation. In the crystal lattice, each mol­ecule of the complex is attached to six mol­ecules of pyrazine, while each pyrazine is connected with two water mol­ecules of the host complexes, leading to the formation of a three-dimensional network (Fig. 2 ▸).

## Synthesis and crystallization   

Crystals of the title compound were obtained by the slow-diffusion method between three layers, the first layer being a solution of [Fe(OTs)_2_]·6H_2_O (0.096 g, 0.2 mmol) and NH_4_SCN (0.046 g, 0.6 mmol) in water (10 ml), the second being a water/methanol mixture (1/1, 10 ml) and the third a solution of 2,3-di­methyl­pyrazine (0.065 g, 0.6 mmol) in methanol (3 ml). After two weeks, red plates grew in the second layer; they were collected, washed with water and dried in air, yield 0.028 g (23%).

## Refinement   

Crystal data, data collection and structure refinement details are summarized in Table 2 ▸. All hydrogen atoms connected to C and O atoms were placed in their expected calculated positions and refined as riding with C—H = 0.98 (CH_3_), 0.95 (C_arom_), O—H = 0.80 (3) Å, and with *U*_iso_(H) = 1.2*U*_iso_(C) with the exception of methyl hydrogen atoms, which were refined with *U*_iso_(H) = 1.5*U*_eq_(C).

## Supplementary Material

Crystal structure: contains datablock(s) global, I. DOI: 10.1107/S2056989015004831/xu5840sup1.cif

Structure factors: contains datablock(s) I. DOI: 10.1107/S2056989015004831/xu5840Isup2.hkl

CCDC reference: 1053032

Additional supporting information:  crystallographic information; 3D view; checkCIF report

## Figures and Tables

**Table 1:** Hydrogen-bond geometry (, )

*D*H*A*	*D*H	H*A*	*D* *A*	*D*H*A*
O1H1*A*N2	0.80(3)	1.95(3)	2.745(4)	172(8)

**Table 2:** Experimental details

Crystal data	
Chemical formula	[Fe(NCS)_3_(H_2_O)_3_]3C_6_H_8_N_2_
*M*_r_	608.57
Crystal system, space group	Trigonal, *R*3*c*
Temperature (K)	133
*a*, *c* ()	16.9383(12), 17.6259(13)
*V* (^3^)	4379.5(7)
*Z*	6
Radiation type	Mo *K*
(mm^1^)	0.77
Crystal size (mm)	0.16 0.12 0.1

Data collection	
Diffractometer	Stoe IPDS II
Absorption correction	Numerical (*X-RED*; Stoe Cie, 2002 ▸)
*T*_min_, *T*_max_	0.908, 0.939
No. of measured, independent and observed [*I* > 2(*I*)] reflections	5784, 1903, 1716
*R*_int_	0.058
(sin /)_max_ (^1^)	0.633

Refinement	
*R*[*F* ^2^ > 2(*F* ^2^)], *wR*(*F* ^2^), *S*	0.038, 0.070, 1.07
No. of reflections	1903
No. of parameters	120
No. of restraints	3
H-atom treatment	H atoms treated by a mixture of independent and constrained refinement
_max_, _min_ (e ^3^)	0.27, 0.28
Absolute structure	Flack *x* determined using 685 quotients [(*I* ^+^)(*I* )]/[(*I* ^+^)+(*I* )] (Parsons *et al.*, 2013 ▸)
Absolute structure parameter	0.03(3)

Computer programs: *X-AREA* and *X-RED* (Stoe Cie, 2002 ▸), *SHELXS97* (Sheldrick, 2008 ▸), *SHELXL2014* (Sheldrick, 2015 ▸), *OLEX2* (Dolomanov *et al.*, 2009 ▸) and *publCIF* (Westrip, 2010 ▸).

## supplementary crystallographic information

### Crystal data

**Table:**

[Fe(NCS)_3_(H_2_O)_3_]·3C_6_H_8_N_2_	*F*(000) = 1902
*M**_r_* = 608.57	*D*_x_ = 1.384 Mg m^−^^3^
Trigonal, *R*3*c*	Mo *K*α radiation, λ = 0.71073 Å
*a* = 16.9383 (12) Å	µ = 0.77 mm^−^^1^
*c* = 17.6259 (13) Å	*T* = 133 K
*V* = 4379.5 (7) Å^3^	Block, red
*Z* = 6	0.16 × 0.12 × 0.1 mm

### Data collection

**Table:**

Stoe IPDS II diffractometer	1716 reflections with *I* > 2σ(*I*)
φ scans and ω scans with κ offset	*R*_int_ = 0.058
Absorption correction: numerical (*X-RED*; Stoe & Cie, 2002)	θ_max_ = 26.8°, θ_min_ = 2.4°
*T*_min_ = 0.908, *T*_max_ = 0.939	*h* = −18→21
5784 measured reflections	*k* = −21→15
1903 independent reflections	*l* = −18→22

### Refinement

**Table:**

Refinement on *F*^2^	Secondary atom site location: difference Fourier map
Least-squares matrix: full	Hydrogen site location: inferred from neighbouring sites
*R*[*F*^2^ > 2σ(*F*^2^)] = 0.038	H atoms treated by a mixture of independent and constrained refinement
*wR*(*F*^2^) = 0.070	*w* = 1/[σ^2^(*F*_o_^2^) + (0.0297*P*)^2^] where *P* = (*F*_o_^2^ + 2*F*_c_^2^)/3
*S* = 1.07	(Δ/σ)_max_ < 0.001
1903 reflections	Δρ_max_ = 0.27 e Å^−^^3^
120 parameters	Δρ_min_ = −0.28 e Å^−^^3^
3 restraints	Absolute structure: Flack *x* determined using 685 quotients [(*I*^+^)-(*I*^-^)]/[(*I*^+^)+(*I*^-^)] (Parsons *et al.*, 2013)
Primary atom site location: structure-invariant direct methods	Absolute structure parameter: −0.03 (3)

### Special details

**Table:**

Geometry. All e.s.d.'s (except the e.s.d. in the dihedral angle between two l.s. planes) are estimated using the full covariance matrix. The cell e.s.d.'s are taken into account individually in the estimation of e.s.d.'s in distances, angles and torsion angles; correlations between e.s.d.'s in cell parameters are only used when they are defined by crystal symmetry. An approximate (isotropic) treatment of cell e.s.d.'s is used for estimating e.s.d.'s involving l.s. planes.

### Fractional atomic coordinates and isotropic or equivalent isotropic displacement parameters (Å^2^)

**Table:**

	*x*	*y*	*z*	*U*_iso_*/*U*_eq_	
Fe1	0.3333	0.6667	0.99891 (7)	0.0201 (2)	
S1	0.29300 (8)	0.86373 (8)	1.16318 (7)	0.0300 (3)	
N1	0.2860 (3)	0.7347 (3)	1.0611 (2)	0.0297 (9)	
O1	0.2256 (2)	0.62277 (19)	0.92686 (19)	0.0221 (6)	
C1	0.2893 (3)	0.7891 (3)	1.1041 (3)	0.0238 (9)	
N2	0.0562 (2)	0.5854 (2)	0.9750 (2)	0.0250 (8)	
N3	−0.1220 (2)	0.5016 (2)	1.0276 (2)	0.0254 (8)	
C2	0.0336 (3)	0.5471 (3)	1.0439 (3)	0.0299 (10)	
H2	0.0796	0.5481	1.0753	0.036*	
C3	−0.0543 (3)	0.5064 (3)	1.0704 (3)	0.0288 (10)	
H3	−0.0674	0.4812	1.1201	0.035*	
C4	−0.1008 (3)	0.5385 (3)	0.9586 (2)	0.0235 (9)	
C5	−0.0099 (3)	0.5815 (3)	0.9318 (3)	0.0229 (9)	
C6	−0.1766 (3)	0.5322 (3)	0.9107 (3)	0.0322 (10)	
H6A	−0.2345	0.4977	0.9379	0.048*	
H6B	−0.1795	0.5010	0.8630	0.048*	
H6C	−0.1655	0.5936	0.8996	0.048*	
C7	0.0144 (3)	0.6246 (3)	0.8553 (3)	0.0304 (10)	
H7A	0.0795	0.6484	0.8459	0.046*	
H7B	0.0012	0.6747	0.8531	0.046*	
H7C	−0.0214	0.5791	0.8166	0.046*	
H1A	0.179 (3)	0.617 (5)	0.943 (4)	0.080*	
H1B	0.206 (5)	0.572 (3)	0.912 (4)	0.080*	

### Atomic displacement parameters (Å^2^)

**Table:**

	*U*^11^	*U*^22^	*U*^33^	*U*^12^	*U*^13^	*U*^23^
Fe1	0.0197 (3)	0.0197 (3)	0.0209 (5)	0.00986 (13)	0.000	0.000
S1	0.0320 (6)	0.0293 (5)	0.0316 (6)	0.0175 (5)	0.0009 (5)	−0.0061 (5)
N1	0.030 (2)	0.031 (2)	0.029 (2)	0.0157 (18)	0.0031 (17)	−0.0024 (18)
O1	0.0180 (14)	0.0201 (14)	0.0283 (17)	0.0096 (13)	−0.0011 (13)	−0.0021 (13)
C1	0.022 (2)	0.030 (2)	0.022 (2)	0.0141 (18)	0.0019 (17)	0.0053 (18)
N2	0.0234 (18)	0.0234 (17)	0.028 (2)	0.0119 (15)	−0.0004 (16)	−0.0026 (15)
N3	0.0236 (17)	0.0233 (17)	0.029 (2)	0.0113 (15)	0.0027 (15)	0.0001 (15)
C2	0.027 (2)	0.037 (2)	0.030 (3)	0.019 (2)	−0.0040 (19)	−0.001 (2)
C3	0.032 (2)	0.029 (2)	0.028 (3)	0.018 (2)	0.0022 (19)	0.0030 (19)
C4	0.021 (2)	0.023 (2)	0.027 (2)	0.0113 (16)	0.0008 (18)	−0.0022 (18)
C5	0.023 (2)	0.0203 (19)	0.027 (2)	0.0121 (17)	0.0023 (17)	−0.0028 (17)
C6	0.027 (2)	0.036 (3)	0.035 (3)	0.017 (2)	−0.002 (2)	−0.002 (2)
C7	0.026 (2)	0.037 (2)	0.028 (3)	0.016 (2)	0.0020 (19)	0.0020 (19)

### Geometric parameters (Å, º)

**Table:**

Fe1—N1	2.025 (4)	N3—C4	1.333 (6)
Fe1—N1^i^	2.025 (4)	C2—H2	0.9500
Fe1—N1^ii^	2.025 (4)	C2—C3	1.372 (6)
Fe1—O1	2.034 (3)	C3—H3	0.9500
Fe1—O1^i^	2.034 (3)	C4—C5	1.415 (6)
Fe1—O1^ii^	2.034 (3)	C4—C6	1.495 (6)
S1—C1	1.615 (5)	C5—C7	1.491 (6)
N1—C1	1.172 (6)	C6—H6A	0.9800
O1—H1A	0.80 (3)	C6—H6B	0.9800
O1—H1B	0.80 (3)	C6—H6C	0.9800
N2—C2	1.339 (6)	C7—H7A	0.9800
N2—C5	1.329 (5)	C7—H7B	0.9800
N3—C3	1.340 (6)	C7—H7C	0.9800
			
N1—Fe1—N1^i^	93.42 (17)	N2—C2—C3	121.9 (4)
N1—Fe1—N1^ii^	93.42 (17)	C3—C2—H2	119.0
N1^i^—Fe1—N1^ii^	93.42 (16)	N3—C3—C2	121.2 (5)
N1—Fe1—O1^ii^	90.67 (14)	N3—C3—H3	119.4
N1^ii^—Fe1—O1^ii^	90.47 (14)	C2—C3—H3	119.4
N1^ii^—Fe1—O1^i^	90.67 (14)	N3—C4—C5	120.9 (4)
N1^i^—Fe1—O1^ii^	174.17 (17)	N3—C4—C6	117.6 (4)
N1—Fe1—O1	90.47 (14)	C5—C4—C6	121.5 (4)
N1^i^—Fe1—O1^i^	90.47 (14)	N2—C5—C4	120.5 (4)
N1^ii^—Fe1—O1	174.17 (17)	N2—C5—C7	118.3 (4)
N1^i^—Fe1—O1	90.67 (14)	C4—C5—C7	121.2 (4)
N1—Fe1—O1^i^	174.17 (17)	C4—C6—H6A	109.5
O1—Fe1—O1^ii^	85.15 (14)	C4—C6—H6B	109.5
O1^ii^—Fe1—O1^i^	85.15 (14)	C4—C6—H6C	109.5
O1—Fe1—O1^i^	85.15 (14)	H6A—C6—H6B	109.5
C1—N1—Fe1	157.0 (4)	H6A—C6—H6C	109.5
Fe1—O1—H1A	118 (6)	H6B—C6—H6C	109.5
Fe1—O1—H1B	115 (6)	C5—C7—H7A	109.5
H1A—O1—H1B	98 (7)	C5—C7—H7B	109.5
N1—C1—S1	179.5 (4)	C5—C7—H7C	109.5
C5—N2—C2	117.7 (4)	H7A—C7—H7B	109.5
C4—N3—C3	117.7 (4)	H7A—C7—H7C	109.5
N2—C2—H2	119.0	H7B—C7—H7C	109.5
			
N2—C2—C3—N3	1.5 (7)	C3—N3—C4—C6	179.4 (4)
N3—C4—C5—N2	0.4 (6)	C4—N3—C3—C2	−0.7 (7)
N3—C4—C5—C7	−178.7 (4)	C5—N2—C2—C3	−1.2 (6)
C2—N2—C5—C4	0.3 (6)	C6—C4—C5—N2	−179.2 (4)
C2—N2—C5—C7	179.5 (4)	C6—C4—C5—C7	1.7 (6)
C3—N3—C4—C5	−0.2 (6)		

Symmetry codes: (i) −*y*+1, *x*−*y*+1, *z*; (ii) −*x*+*y*, −*x*+1, *z*.

### Hydrogen-bond geometry (Å, º)

**Table:**

*D*—H···*A*	*D*—H	H···*A*	*D*···*A*	*D*—H···*A*
O1—H1*A*···N2	0.80 (3)	1.95 (3)	2.745 (4)	172 (8)
